# Sympatho‐excitatory response to pulmonary chemosensitive spinal afferent activation in anesthetized, vagotomized rats

**DOI:** 10.14814/phy2.13742

**Published:** 2018-06-14

**Authors:** Julia Shanks, Zhiqiu Xia, Steven J. Lisco, George J. Rozanski, Harold D. Schultz, Irving H. Zucker, Han‐Jun Wang

**Affiliations:** ^1^ Department of Cellular and Integrative Physiology University of Nebraska Medical Center Omaha Nebraska; ^2^ Department of Anesthesiology University of Nebraska Medical Center Omaha Nebraska

**Keywords:** Blood pressure, cardiovascular reflexes, pulmonary diseases, sympathetic nerve activity

## Abstract

The sensory innervation of the lung is well known to be innervated by nerve fibers of both vagal and sympathetic origin. Although the vagal afferent innervation of the lung has been well characterized, less is known about physiological effects mediated by spinal sympathetic afferent fibers. We hypothesized that activation of sympathetic spinal afferent nerve fibers of the lung would result in an excitatory pressor reflex, similar to that previously characterized in the heart. In this study, we evaluated changes in renal sympathetic nerve activity (RSNA) and hemodynamics in response to activation of TRPV1‐sensitive pulmonary spinal sensory fibers by agonist application to the visceral pleura of the lung and by administration into the primary bronchus in anesthetized, bilaterally vagotomized, adult Sprague‐Dawley rats. Application of bradykinin (BK) to the visceral pleura of the lung produced an increase in mean arterial pressure (MAP), heart rate (HR), and RSNA. This response was significantly greater when BK was applied to the ventral surface of the left lung compared to the dorsal surface. Conversely, topical application of capsaicin (Cap) onto the visceral pleura of the lung, produced a biphasic reflex change in MAP, coupled with increases in HR and RSNA which was very similar to the hemodynamic response to epicardial application of Cap. This reflex was also evoked in animals with intact pulmonary vagal innervation and when BK was applied to the distal airways of the lung via the left primary bronchus. In order to further confirm the origin of this reflex, epidural application of a selective afferent neurotoxin (resiniferatoxin, RTX) was used to chronically ablate thoracic TRPV1‐expressing afferent soma at the level of T1–T4 dorsal root ganglia pleura. This treatment abolished all sympatho‐excitatory responses to both cardiac and pulmonary application of BK and Cap in vagotomized rats 9–10 weeks post‐RTX. These data suggest the presence of an excitatory pulmonary chemosensitive sympathetic afferent reflex. This finding may have important clinical implications in pulmonary conditions inducing sensory nerve activation such as pulmonary inflammation and inhalation of chemical stimuli.

## Introduction

In a wide range of physiological and pathophysiological conditions, the heart and lungs work in synergy in order to maintain adequate oxygenation of tissues (Coleridge and Coleridge 1994; Mazzone and Undem 2016). The majority of research to date has focused on the role of pulmonary vagal neurons on sensing and regulating respiratory and upper airway function (Bergren and Peterson 1993). The vagus is a mixed nerve containing afferents that consist of fast conducting myelinated A fibers, slow conducting unmyelinated C fibers, and parasympathetic cholinergic efferent fibers. Stimulation of vagal sensory fibers has been shown to regulate rate and depth of breathing, basal tidal volume via the Herring–Breuer reflex (Carr and Undem 2003) (Paintal 1973) (Widdicombe and Lee 2001), cough (Canning and Chou 2009; Taylor‐Clark 2015), and innervation of neuroepithelial bodies (Chang et al. 2015). The bradycardia observed in response to lung inflation (Shepherd 1981) and to inhalation of noxious stimuli (Hazari et al. 2011; Hooper et al. 2016) is thought to be mediated by afferents of vagal origin.

Although the traditional view has been that vagal‐derived afferents are the predominant sensory fibers within the lung, a number of studies have alluded to the presence of pulmonary spinal afferents using immunostaining (Nonomura et al. 2017) and retrograde labeling (Springall et al. 1987; Kummer et al. 1992). However, the exact role and function of spinal afferent neurons in the lung is still unknown. Previous studies have indicated pressor, depressor, and biphasic blood pressure responses to cardiopulmonary spinal afferent activation (Weaver 1977; Kostreva et al. 1981; Wang et al. 2003; Soukhova‐O'Hare et al. 2006; Hooper et al. 2016). The differences in whether an excitatory or inhibitory blood pressure response was observed in these studies are likely due to the preparations and experimental protocols used. Therefore, to date, the effect of specific pulmonary spinal afferent activation on cardiovascular regulation remains inconclusive. In this study, we hypothesized that similar to the sympathetic afferent cardiac pressor and exercise pressor reflexes of spinal origin in the rat, an excitatory pulmonary spinal pressor reflex exists when lung visceral pleura spinal afferent endings are stimulated with physiological and pharmacological TRPV1 agonists. We aimed to study the location of chemosensitive sympathetic spinal afferents within the dorsal and ventral lung surfaces, as well as internally within the lung via bronchial administration of agonist in vagotomized rats. The effects of activation of the spinal pulmonary chemosensitive reflex on cardiovascular function and RSNA in both vagotomized and nonvagotomized rats were measured. The spinal origin of these afferents was studied by activating lung surface nerve terminals with and without denervation of TRPV1 afferent neurons in the thoracic dorsal root ganglia (DRG).

## Methods

### Animal models

Experiments were performed on male Sprague‐Dawley (SD) rats weighting 300–400 g (Charles River, USA). These experiments were approved by the Institutional Animal Care and Use Committee of the University of Nebraska Medical Center and carried out under the guidelines of the National Institutes of Health *Guide for the Care and Use of Laboratory Animals*, and complies with the animal ethics guidelines or the Journal of Physiology as outline in the editorial by Grundy (2015). Animals were housed in an on‐site facility and were allowed to acclimate for at least 1 week following arrival to their new environment. Water and laboratory rat chow were provided ad libitum, and animals were housed in 12 h light/dark cycles. For chronic surgical procedures, isoflurane (2%) was administered for induction and maintenance of anesthetic, analgesia of bupenex (0.05 mg/kg) was given on the day of surgery, and carprofen (5 mg/kg) for 3 days postsurgery as postprocedure pain management. An overdose of pentobarbital (150 mg/kg) was used for rat euthanasia as approved by the supervising veterinarian and the Panel on Euthanasia of the American Veterinary Medical Association. Euthanasia was confirmed by cervical dislocation and removal of vital organs.

### General surgical preparation for acute experiments

For the acute terminal experiments, rats were anesthetized with urethane (800 mg/kg ip) and *α*‐chloralose (40 mg/kg ip). Anesthetic plane was monitored by establishing rats were unresponsives to pedal withdrawal and corneal reflexes. The trachea was cannulated, and rats were ventilated artificially with room air supplemented with oxygen (~40% O_2_). A Millar catheter (SPR 524; size, 3.5‐Fr; Millar Instruments, Houston, TX) was advanced through the right common carotid artery and progressed into the aorta and left in place to record arterial pressure (AP). Mean arterial pressure (MAP) and heart rate (HR) were derived from the arterial pressure pulse using Chart 7.1 software and an analog to digital converter (PowerLab model 16S; AD Instruments, Colorado Springs, CO). The right jugular vein was cannulated for intravenous injections and administration of saline at a rate of 3 mL/h. Body temperature was maintained between 37 and 38°C by a heating pad. In most of the experiments, the cervical vagal nerves were cut bilaterally to prevent any reflex responses observed from vagal afferent activation, so as to observe only responses related to pulmonary spinal afferent activation. In some experiments, the vagal nerves were left intact in order to determine whether the pulmonary pressor reflex was still present with intact pulmonary vagal innervation.

### Recording renal sympathetic nerve activity

In acute surgical preparations described above, RSNA was recorded as previously described (Gao et al. 2005; Wang et al. 2010; Becker et al. 2016). In brief, the left kidney, renal artery, and nerves were exposed through a left retroperitoneal flank incision. Sympathetic nerves running on or beside the renal artery were identified. The renal sympathetic nerves were placed on a pair of platinum–iridium recording electrodes and cut distally to avoid recording afferent activity. Nerve activity was amplified (×10,000) and filtered (bandwidth: 100 to 3000 Hz) using a Grass P55C preamplifier. The nerve signal was displayed on a computer where it was rectified, integrated, sampled (1 KHz), and converted to a digital signal by the PowerLab data acquisition system. At the end of the experiment, the rat was euthanized with an overdose of pentobarbital sodium. Respective noise levels were subtracted from the nerve recording data before percent changes from baseline were calculated. Integrated RSNA (iRSNA) was normalized as 100% of mean baseline during the control period (Becker et al. 2016).

### Activation of cardiac or pulmonary spinal afferents

Epicardial application of capsaicin (Cap) and bradykinin (BK) has been demonstrated to effectively stimulate cardiac spinal afferents via the TRPV1 receptor (Zahner et al. 2003) and the bradykinin receptor 2 (BKR2), respectively (Soukhova‐O'Hare et al. 2006; Mazzone and Undem 2016). Therefore, a similar approach was employed to activate cardiac or pulmonary spinal afferents in this study. The chest was opened through the fourth intercostal space. A square of filter paper (3 × 3 mm) saturated with Cap (10 *μ*g/mL), or BK (1 or 10 *μ*g/mL), was applied randomly to the ventral or dorsal surface of the left lung, distant from the hilum. In order to prevent the agonist from activating cardiac spinal afferents being absorbed into the main pulmonary vessels, special attention was paid to avoid placing the filter paper too close to the hilum. Hemodynamics and RSNA were continuously recorded. After the responses peaked, the lung was rinsed three times with 10 mL of warm normal saline. Thirty minutes were allowed to elapse between subsequent stimulations, to allow MAP, HR, and RSNA to return and stabilize at control levels. After the ventral and dorsal lung applications, we also applied BK and Cap onto the surface of left ventricular free wall of the heart in the same vagotomized rats to investigate whether both agents evoked similar hemodynamic and neural responses from the heart.

Intrabronchial application of BK was performed by administering 100 *μ*L of saline to test any affect of vehicle administration, followed by a bolus injection of 100 *μ*L of BK 10 *μ*g/mL, into the left primary bronchus by a specially adapted ventilation tube that was positioned at the opening of the primary bronchus, with catheter progressed 2 mm into the left primary bronchus, this allowed for the animal to be continuously ventilated while the drug was being applied. One hour was allowed between applications of saline and BK. Intrabronchial experiments were performed in a separate group of animals because it was not possible to “wash” the drug out, and it could not be confirmed that the drug would no longer be active within the lung, potentially compounding repeated drug applications in the animal.

### Upper thoracic spinal sympathetic afferent denervation

In some rats, the upper thoracic spinal sympathetic afferents were chronically ablated prior to study. Briefly, rats were anesthetized using 2–3% isoflurane:oxygen mixture. Rats were placed in the prone position, and a small midline incision was made in the region of the T13–L1 thoracic vertebrae. Following dissection of the superficial muscles, two small holes (approximately 2 × 2 mm) were made in the left and right sides of T13 vertebrae. A polyethylene catheter (PE‐10) was inserted into the subarachnoid space via one hole and gently advanced about 4 cm approximating the T1 level. The upper thoracic sympathetic afferent ganglia were ablated by injecting resiniferatoxin (RTX; Sigma‐Aldrich), an ultrapotent agonist of the TRPV1 receptor into the subarachnoid space via the catheter. RTX has been shown to ablate the TRPV1‐positive spinal afferent nerve endings on the heart (Wang et al. 2014, 2017). RTX (1 mg; Sigma‐Aldrich) was dissolved in a 1:1:8 mixture of ethanol, Tween‐80 (Sigma‐Aldrich), and isotonic saline. The first injection of RTX (6 *μ*g/mL, 10 *μ*L) was made at a very slow speed (~1 min) to minimize the diffusion of the drug. The catheter was then pulled back to T2, T3, and T4, respectively, to perform serial injections (10 *μ*L/each) at each segment. The catheter was withdrawn, and the same injections were repeated on the other side. Silicone gel was used to seal the hole in the T13 vertebra. The skin overlying the muscle was closed with a 3‐0 polypropylene simple interrupted suture, and betadine was applied to the wound. For postprocedure pain management, buprenorphine (0.05 mg/kg) was subcutaneously injected immediately after surgery and twice daily for 2 days. Terminal experiments were carried out 9‐10 weeks post‐TRPV1 neuronal ablation.

### Immunofluorescence labeling of TRPV1 receptors in thoracic dorsal root ganglia and spinal cord

To confirm that epidural T1–T4 application of RTX successfully ablated the TRPV1‐expressing DRG neurons, immunofluorescence labeling experiments were conducted on the T1–T4 DRGs and spinal cord. At the end of the study, rats (*n* = 3/each group) were anesthetized with pentobarbital sodium (40 mg/kg, i.p.), and perfused through the aorta, first with 100 mL heparinized saline followed by 500 mL 4% paraformaldehyde (in 0.1 mol/L sodium phosphate buffer, PBS, pH = 7.4). The thoracic DRGs and spinal cord were immediately removed and immersed in the 4% paraformaldehyde solution overnight at 4°C. The tissues were then transferred to 30% sucrose in PBS and kept in the solution until they sank to the bottom. DRGs were sectioned at 14 *μ*m, and the spinal cord at 30 *μ*m on a Leica cryostat (−20°C) and thawed onto gelatin‐coated slides.

The double immunostaining of TRPV1 receptors with isolectin IB4 (a C‐fiber neuronal marker) in DRGs (Wang et al. 2013) and triple immunostaining of TRPV1 receptors with the isolectin IB4 and substance P (SP, an peptidergic C‐fiber neuron marker), in ganglionic or spinal cord sections, was performed by pre‐incubation of 10% goat serum for 60 min, prior to incubation with rabbit anti‐TRPV1 antibody (1:200 dilution, NB100‐1617, Novus Biologicals, Littleton, CO, USA) and/or mouse anti‐SP antibody (1:200 dilution, sc‐58591, Santa Cruz, Inc, Dallas, TX USA) overnight at 4°C. Sections were then washed with PBS and incubated with fluorescence‐conjugated secondary antibody (Alexa 488‐conjugated goat anti‐rabbit IgG and pacific blue‐conjugated goat anti‐mouse IgG, 1:200, Invitrogen, CA, USA) and Alexa Fluor R 568 conjugated isolectin‐B4 (1:200, Invitrogen, CA, USA) for 60 min at room temperature. Slides were observed on a Leica fluorescent microscope, and images captured using a digital camera system. No staining was seen when a negative control was performed with PBS instead of the primary antibody (data not shown).

### Chemicals and reagents

RTX, Cap, and BK were purchased from Sigma. RTX and Cap were dissolved in a 1:1:8 mixture of ethanol, Tween‐80 (Sigma‐Aldrich), and isotonic saline (50 *μ*g/mL). RTX was diluted to a working concentration of 6 *μ*g/mL, and Cap was diluted to 10 *μ*g/mL using sterile saline prior to administration. Bradykinin was dissolved in sterile saline with 100 *μ*g/mL in stock and was further diluted in sterile saline to the working concentration (10 *μ*g/mL), when used.

### Statistical analysis

Statistical analysis was designed to test the hypotheses that the application of agonists to the surface of the lung would cause a change in hemodynamic parameters (MAP, HR), and RSNA compared to application of saline to the lung surface, or postspinal sympathetic ablation with RTX. Treatments were randomized between experiments so that agonists were not applied in the same order in each group. RTX, vagal intact, and bronchial application of agonists were each performed in a separate set of animals. Number of samples “*n*” equals the number of animals in each group.

Statistics were analyzed using GraphPad Prism. Differences between treatments were determined by a one‐way ANOVA followed by the Dunnett's post hoc test to correct for multiple comparisons. All values are expressed as mean ± SE of the mean (SEM). *P* < 0.05 was considered statistically significant. Normality of data sets was confirmed using the D'Agostine and Pearson normality test. Baseline data are reported in Tables 1, 2, 3, 4, 5, 6.

**Table 1:** Topical application of BK on lung causes a sympatho‐excitatory response in vagotomized rats

	Vehicle (*n* = 6)		Ventral BK 1.0 (*n* = 6)		Ventral BK10 (*n* = 11)		Dorsal BK10 (*n* = 7)	
	Baseline	∆ Change	Baseline	∆ Change	Baseline	∆ Change	Baseline	∆ Change
MAP (mmHg)	93.6 ± 3.9	0.8 ± 0.5	98.8 ± 6.2	14.7 ± 3.1^a^	89.2 ± 2.7	19.5 ± 2.1^a^	89.1 ± 4.1	11.6 ± 2.4^a^
HR (bpm)	385.7 ± 12.7	1.7 ± 0.9	355.5 ± 16.8	18.2 ± 2.7^a^	368.6 ± 12.0	22.4 ± 3.0^a^	367.3 ± 18.6	12.3 ± 2.5^a^
RSNA (%baseline)	100%	1.5 ± 1.1	100%	58.0 ± 12.7^a^	100%	88.2 ± 7.2^a^	100%	44.6 ± 8.4^a^

Values are mean ± SE. MAP, mean arterial pressure; HR, heart rate; RSNA, renal sympathetic nerve activity.

a *P* < 0.05 vs. vehicle.

**Table 2:** Topical left lung visceral pleura application of capsaicin (Cap) causes a sympatho‐excitatory response in vagotomized rats

	Vehicle		Ventral Cap 10				Dorsal Cap 10			
	(*n* = 6)		Depressor (*n* = 7)		Pressor (*n* = 10)		Depressor (*n* = 5)		Pressor (*n* = 5)	
	Baseline	∆ Change	Baseline	∆ Change	Baseline	∆ Change	Baseline	∆ Change	Baseline	∆ Change
MAP (mmHg)	93.6 ± 3.9	0.8 ± 0.5	92.7 ± 5.0	−15.0 ± 2.2^a^	85.8 ± 6.5	13.9 ± 1.9^a^	88.4 ± 6.0	−20.0 ± 3.4^a^	84.8 ± 6.5	7.6 ± 1.9
HR (bpm)	385.7 ± 12.7	1.7 ± 0.9	379 ± 12.3	0.7 ± 1.6	394 ± 14.1	15.4 ± 3.2^a^	388.2 ± 12.9	−1.2 ± 1.0	381.5 ± 10.1	10.8 ± 1.5
RSNA (%.baseline)	100%	1.5 ± 1.1	100%	41.0 ± 4.4	100%	78.1 ± 12.4^a^	100%	41.1 ± 23.8	100%	74.0 ± 18.8^a^

Values are mean ± SE. MAP, mean arterial pressure; HR, heart rate; RSNA, renal sympathetic nerve activity.

a *P *< 0.05 vs. vehicle.

**Table 3:** Epicardial application of bradykinin (BK) and capsaicin (Cap) causes a sympatho‐excitatory response in vagotomized rats

	Heart vehicle		Heart BK10		Heart Cap 10			
	(*n* = 6)		(*n* = 8)		Depressor (*n* = 5)		Pressor (*n* = 8)	
	Baseline	Δ Change	Baseline	Δ Change	Baseline	Δ Change	Baseline	Δ Change
MAP (mmHg)	95.2 ± 5.9	0.6 ± 0.4	84.6 ± 3.2	18.6 ± 1.6^a^	95.8 ± 5.2	−16.2 ± 2.4^a^	89.8 ± 4.4	18.1 ± 1.4^a^
HR (bpm)	335.5 ± 21.1	0.6 ± 1.0	369.8 ± 7.8	22.9 ± 2.7^a^	368.6 ± 5.9	2.2 ± 12^a^	365.1 ± 6.5	17.9 ± 0.9^a^
RSNA (%.baseline)	100%	0.3 ± 0.8	100%	104.3 ± 10.1^a^	100%	91.1 ± 11.6^a^	100%	113.0 ± 9.6^a^

Values are mean ± SE. MAP, mean arterial pressure; HR, heart rate; RSNA, renal sympathetic nerve activity.

a *P*<0.05 vs. vehicle.

**Table 4:** Topical left lung visceral pleura application of bradykinin (BK) causes a sympatho‐excitatory response in nonvagotomized rats

	Vehicle		Vehicle BK10		Vehicle Cap 10			
	(*n* = 4)		(*n* = 9)		Depressor (*n* = 6)		Pressor (*n* = 8)	
	Baseline	Δ Change	Baseline	Δ Change	Baseline	Δ Change	Baseline	Δ Change
MAP (mmHg)	73.5 ± 8.5	0.5 ± 2.4	85 ± 15.9	14.9 ± 4.4^a^	89.6 ± 17.6	−8.7 ± 3.0^a^	85.9 ± 17.5	10.0 ± 6.2^a^
HR (bpm)	335 ± 21.2	2.2 ± 3.7	347.0 ± 30.4	21.7 ± 7.9^a^	345.2 ± 42.79	7.4 ± 6.6^a^	349.3 ± 38.5	25.8 ± 12.0^a^
RSNA (%.baseline)	100%	1.8 ± 5.78	100%	93.8 ± 36.2^a^	100%	38.6 ± 31.9^a^	100%	68.4 ± 33.6 a

Values are mean ± SE. MAP, mean arterial pressure; HR, heart rate; RSNA, renal sympathetic nerve activity.

a *P* < 0.05 vs. vehicle.

**Table 5:** Intrabronchial application of bradykinin (BK) causes a sympatho‐excitatory response in vagotomized rats

	Vehicle		BK10	
	(*n* = 4)		(*n* = 6)	
	Baseline	Δ Change	Baseline	Δ Change
MAP (mmHg)	77.1 ± 3.9	−1.8 ± 2.5	80.7 ± 19.8	12.1 ± 3.4^a^
HR (bpm)	367.5 ± 73.3	−1.8 ± 2.6	361.4 ± 58.4	19.1 ± 4.6^a^
RSNA (%baseline)	100%	−0.5 ± 3.8	100%	68.4 ± 19.5^a^

Values are mean ± SE. MAP, mean arterial pressure; HR, heart rate; RSNA, renal sympathetic nerve activity.

a *P* < 0.05 vs. vehicle.

**Table 6:** T1–T4 epidural application of RTX abolishes the sympatho‐excitatory response to both cardiac and pulmonary spinal afferent stimulation

	Heart				Lung			
	Cap 10 (*n* = 5)		BK 10 (*n* = 5)		Cap 10 (*n* = 9)		BK 10 (*n* = 9)	
	Baseline	Δ Change	Baseline	Δ Change	Baseline	Δ Change	Baseline	Δ Change
MAP (mmHg)	95.0 ± 5.2	1.8 ± 0.9	95.6 ± 6.5	2.2 ± 1.0^a^	101.2 ± 6.7	−0.2 ± 1.2	95.6 ± 4.6	2.6 ± 0.8
HR (bpm)	301.6 ± 5.0	3.0 ± 0.9	308.3 ± 16.3	3.2 ± 0.9^a^	308.0 ± 9.8	−3.8 ± 3.1	308.3 ± 11.4	3.4 ± 1.4
RSNA (%baseline)	100%	7.8 ± 1.8	100%	7.3 ± 1.6^a^	100%	5.8 ± 2.8	100%	9.3 ± 4.3

Values are mean ± SE. MAP, mean arterial pressure; HR, heart rate; RSNA, renal sympathetic nerve activity.

a *P*<0.05 vs. vehicle.

## Results

### Pulmonary application of bradykinin in vagotomized rats

**Figure 1:**
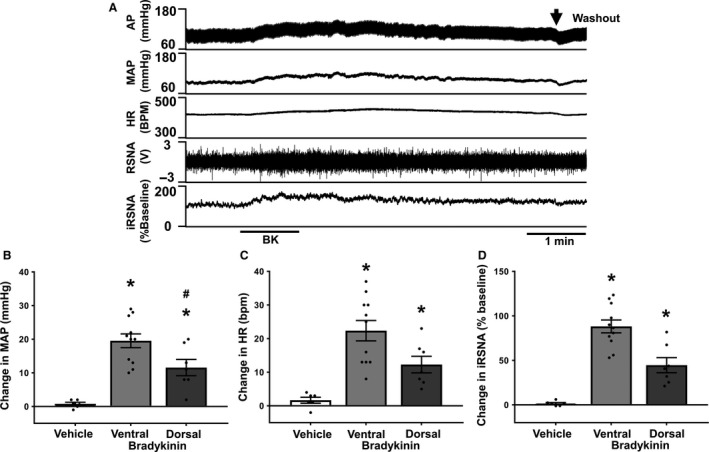
(A) representative recording showing the response to topical application of bradykinin (BK) (10 *μ*g/mL) on to the ventral surface of the lung in an anesthetized and vagotomized rat. Arterial blood pressure (AP), mean arterial blood pressure (MAP), heart rate (HR), renal sympathetic nerve activity (RSNA), integrated RSNA (iRSNA) as a % of baseline. (B) Group mean data of the change in MAP from baseline. (C) Group mean data of the change in HR from baseline. (D) Group mean data of the change in RSNA from baseline, (Veh, *n* = 6; BK (10 *μ*g/mL): ventral, *n* = 11; dorsal, *n* = 7). Data presented as mean ± SEM. *P < 0.05 compared to vehicle, ^#^ *P *< 0.05 compared to ventral.

Topical application of BK onto the ventral surface of the lung produced a robust, dose‐dependent increase in MAP, HR, and RSNA in anesthetized, vagotomized rats (Fig. 1 and Table 1). The latency from application of agonist to onset of sympatho‐excitation was very short (<5 sec). The peak increase in RSNA typically occured 20–30 sec after the onset of the response and diminished slowly over time. Increases in both HR and MAP peaked later than RSNA in most experiments (50–60 sec after the onset). Washout of BK from the visceral pleura of the lung caused an immediate reduction in MAP, HR, and RSNA. The rapidity of the initial phase of the sympatho‐excitatory response to topical application of BK suggests that the response was mediated through a local afferent reflex mechanism rather than a systematic effect caused by absorption of BK into systemic circulation. Furthermore, we examined whether differences in the cardiovascular responses to topical BK application existed dependent upon the local of application on the visceral pleura. Interestingly, we found that this sympatho‐excitatory response was significantly greater when BK was selectively applied to the ventral surface of left lung (middle region), compared to the left dorsal surface in the same rat.

### Pulmonary application of capsaicin in vagotomized rats

**Figure 2:**
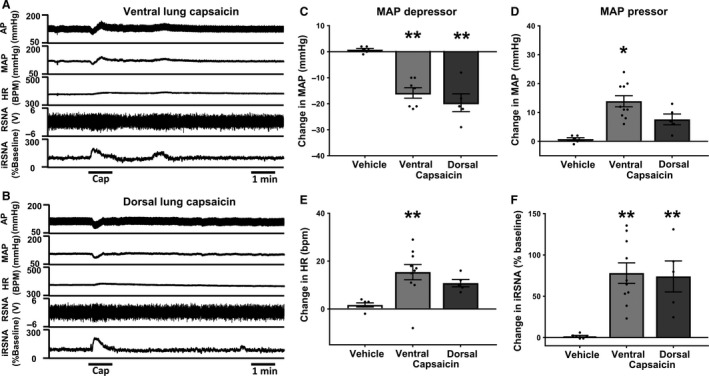
(A) representative recording showing that topical application of capsaicin (Cap) (10 *μ*g/mL) on to the ventral surface of the lung, produces a predominant depressor response. (B) Representative raw data trace showing topical application of Cap (10 *μ*g/mL) on to the dorsal surface of the lung exhibiting a biphasic blood pressure response combined with pure tachycardia and sympatho‐excitation, (A and B) in an anesthetized double‐vagotomized rat. (C–F) Group mean change from baseline, (ventral, depressor *n* = 7, pressor *n* = 10. Dorsal, depressor *n* = 5, pressor *n* = 5.) Data presented as mean ± SEM. *P < 0.05 compared to vehicle, **P < 0.01 compared to vehicle. Abbreviations as in Figure 1.

To selectively stimulate pulmonary spinal TRPV1‐containing afferents, we applied Cap (10 *μ*g/mL), a potent agonist of the TRPV1 receptor, onto the visceral pleura of the lung in anesthetized, bilaterally vagotomized rats. Topical application of Cap produced a biphasic change in blood pressure in most animals, which was different from the response to topical pulmonary application of BK (Fig. 2 and Table 2). The depressor response preceded the pressor response (observed in 7 of 10 animals, Fig. 2A and B). Furthermore, the depressor response was significantly greater with application of Cap to the dorsal lung surface compared to the ventral lung surface, whereas the pressor response was significantly greater with ventral application of Cap compared to dorsal. However, in 3 of 10 animals, we only observed a pure pressor response to topical application of Cap to either side of the lung, indicating the variability in blood pressure regulation that exists when pulmonary TRPV1‐positive afferents are stimulated in these rats. In spite of the variability in AP responses, HR and RSNA always increased during Cap application (Fig. 2 and Table 2), similar to that seen during pulmonary BK application. The latency of the increase in HR and RSNA for Cap was similar to that seen for BK application.

**Figure 3:**
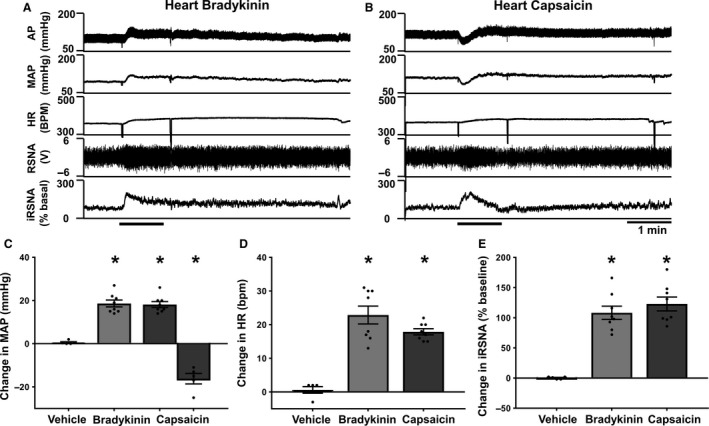
Representative recordings (A and B) and summary data (C–D) showing the effect of epicardial application of BK (A) and Cap (B) (10 *μ*g/mL) on hemodynamic parameters in anesthetized and vagotomized rats. Similar to lung application, epicardial application of BK evoked a pressor response, tachycardia, and sympatho‐excitation, whereas epicardial application of Cap caused similar biphasic AP response in most vagotomized rats (five of eight rats). In three of eight animals, we only observed a pure pressor response to epicardial application of Cap. (*n* = 8 in BK and Cap pressor groups, *n* = 5 in the Cap depressor group.) Data presented as mean ± SEM. *P < 0.05 compared to vehicle. Abbreviations as in Figure 1.

It was also noticed that the differential AP responses between Cap and BK application to the lungs were similar to those observed from applications to the heart. We observed that epicardial application of BK caused a pressor response, whereas epicardial application of Cap caused biphasic AP responses in most vagotomized rats (five of eight rats Fig. 3 and Table 3). In three of eight animals, we only observed a pure pressor response to epicardial application of Cap.

### Pulmonary application of bradykinin and capsaicin in rats with intact vagi

**Figure 4:**
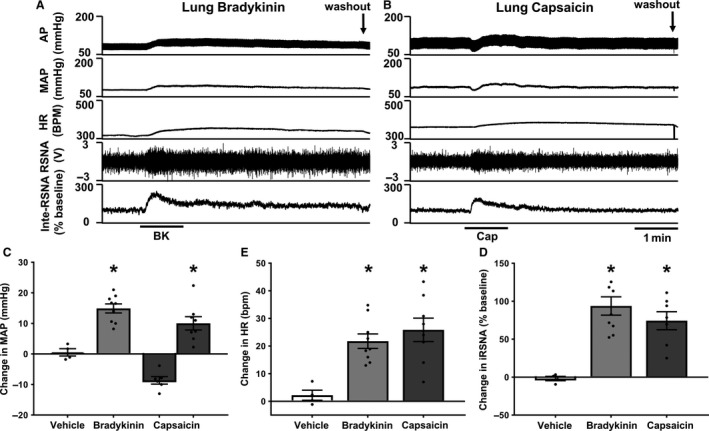
(A) representative recording showing topical application of bradykinin (BK) 10 *μ*g/mL on to the ventral surface of the lung in an anesthetized rat with intact vagus nerve. (B) Representative recording showing topical application of Cap (10* μ*g/mL) on to the ventral surface of the lung in an anesthetized rat with intact vagus nerve. (C–E) Group mean data, change from baseline. (C) MAP (Veh *n* = 4. BK (10 *μ*g/mL) *n* = 9. Cap (10 *μ*g/mL) pressor *n* = 8, depressor *n* = 6.) (D) HR (Veh *n* = 4. BK (10 *μ*g/mL) *n* = 9. Cap (10 *μ*g/mL) pressor *n* = 8.) E, RSNA (Veh *n* = 4. BK (10 *μ*g/mL) *n* = 9. Cap (10 *μ*g/mL) pressor *n* = 8.) Data presented as mean ± SEM. *P < 0.05 compared to vehicle. Abbreviations as in Figure 1.

To establish whether this pulmonary pressor reflex still exists in the presence of pulmonary vagal innervation, experiments were repeated in anesthetized rats with intact vagal nerves. Topical application of BK (10 *μ*g/mL) and Cap (10 *μ*g/mL) produced an increase in MAP, HR, and RSNA compared to vehicle controls when applied to the ventral visceral pleura of the lung (Fig. 4 and Table 4). Cap also produced a depressor response in six of eight animals studied (MAP: Cap 10 *μ*g/mL depressor −8.7 ± 1.2 mmHg), as previously shown in Figure 2A depressor response was only observed in MAP. This demonstrates that a very similar response is observed in animals with intact thoracic vagal innervation as in those animals with bilateral vagotomy.

### Intrabronchial application of bradykinin in vagotomized rats

**Figure 5:**
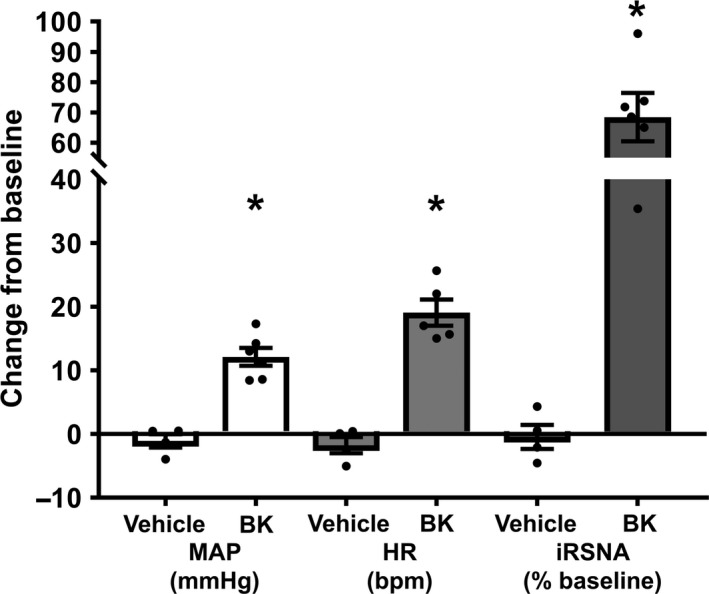
Group mean data representing change from baseline of MAP, HR, and RSNA in response to intrabronchial application of BK or saline vehicle in the anesthetized vagotomized rat (BK *n* = 6, Veh *n* = 4). Data presented as mean ± SEM. *P < 0.05 compared to vehicle. Abbreviations as in Figure 1.

To test the physiological implications of intrabronchial application of BK (acting as and inhaled activator of spinal afferents), BK 10* μ*g/mL or saline vehicle was administered directly into the lung at the level of the primary bronchi through a tracheal cannula in vagotomized, anaesthetized rats. BK produced a significant cardiac pressor response and increase in sympathetic nerve activity compared to vehicle control (Fig. 5 and Table 5). This suggests that the phenomenon observed with pulmonary surface application of BK is reproducible when BK is applied onto the airways.

### Ablation of sympathetic spinal ganglia with RTX abolished neural and hemodynamic changes to cardiac and pulmonary topical application of bradykinin and capsaicin

**Figure 6:**
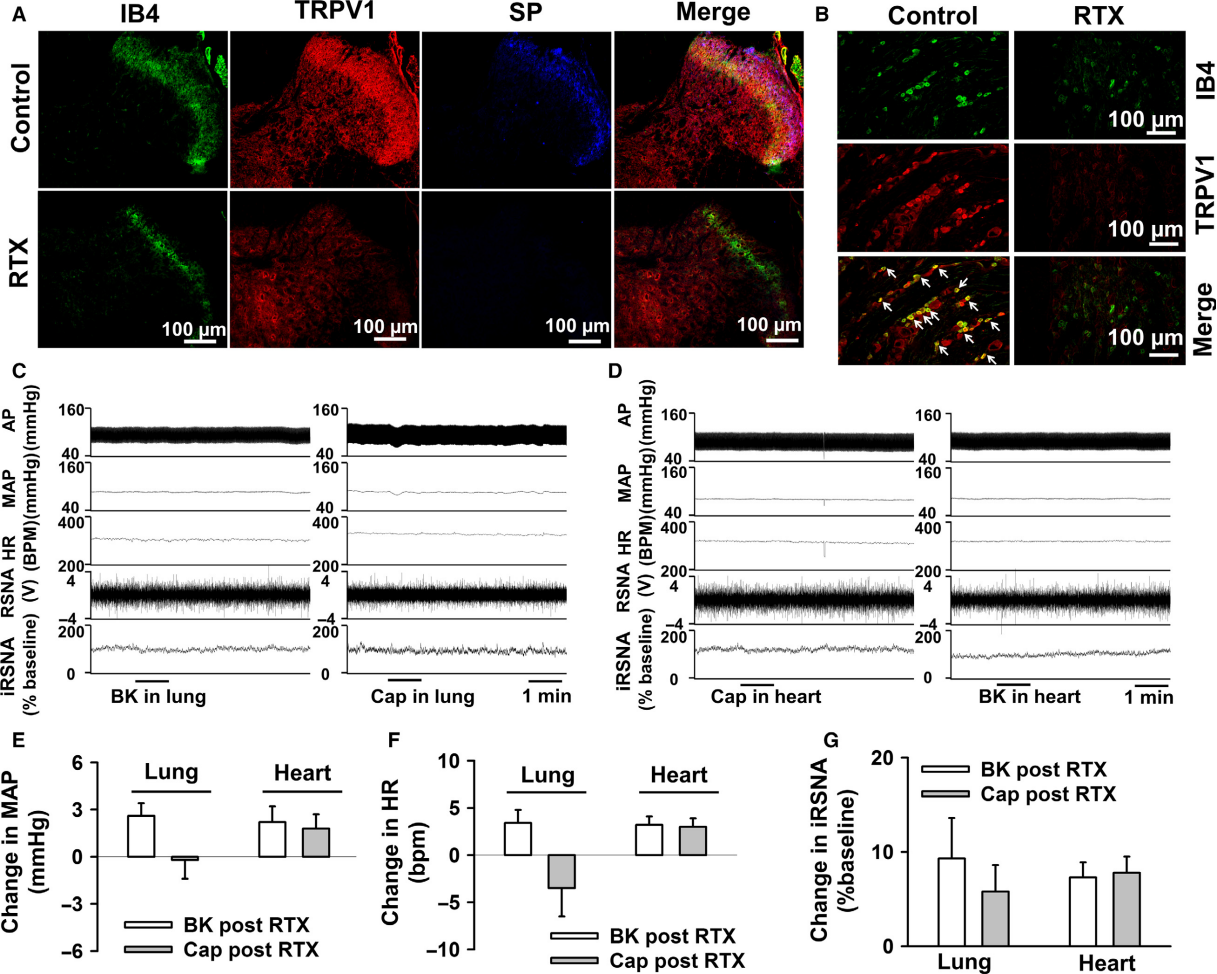
The efficacy of epidural T1–T4 application of RTX in ablating the cardiac/pulmonary sympathetic afferent reflex in rats. (A) Representative images showing that the TRPV1‐positive terminal density at the dorsal horn of T2 spinal cord was largely diminished in RTX‐treated rats, which was associated with both reduced substance P‐positive (Peptidergic C‐fiber marker) and IB4‐positive (nonpeptidergic C‐fiber marker) terminal density at the dorsal horn. (B) Representative images showing that epidural application of RTX largely reduced the number of both TRPV1‐positive and IB4‐positive (a C‐fiber marker) neurons in T2 dorsal root ganglion. (C and D) Representative raw data trace showing topical application of BK (10 *μ*g/mL) and Cap (10 *μ*g/mL) onto the ventral surface of the lung (C, *n* = 9) or heart (D, *n* = 5) in anesthetized, vagotomized rats after pretreatment with T1–T4 epidural RTX to ablate sympathetic spinal afferents. (E–G) Group mean data showing that topical application of BK and Cap onto lung and heart caused very little hemodynamic or neural response in rats with epidural T1–T4 application of RTX. Data presented as mean ± SEM. *P < 0.05 compared to vehicle. Abbreviations as in Figure 1.

In order to confirm the spinal afferent origin of pulmonary BK‐induced sympatho‐excitation, we performed pulmonary spinal afferent denervation by T1–T4 epidural application with the selective afferent neurotoxin RTX. Epidural application of RTX reduced the number of both TRPV1‐positive and IB4‐positive DRG neurons at the thoracic levels (Fig. 6A and B). As a consequence of reduced TRPV1‐positive DRG neurons by RTX application, the TRPV1‐positive terminal density in the dorsal horn of thoracic spinal cord was also diminished in RTX‐treated rats. Considering that the TRPV1 receptor is expressed in both peptidergic (substance P‐positive) and nonpeptidergic (IB4‐positive) DRG soma and nerve terminals, ablation of TRPV1‐positive DRG neurons also caused a reduction in both substance P‐positive and IB4‐positive terminal density in the dorsal horn (Fig. 6A). In functional experiments, we confirmed that epidural application of RTX abolished the pressor and sympatho‐excitatory responses to pulmonary topical application of BK (Fig. 6C–G and Table 6), and abolished both the pressor and the depressor changes in response to Cap (Fig. 6D–G and Table 6) in both the lung and the heart. This indicates that the hemodynamic responses to topical pulmonary and cardiac BK and Cap are due to activation of a spinal afferent pathway.

## Discussion

The key findings of this study are that activation of chemosensitive pulmonary spinal afferent nerve fibers by bradykinin and capsaicin can evoke an increase in RSNA, AP, and HR. This reflex is present in both vagotomized and nonvagotomized anaesthetized rats, and is abolished after chronic cardiopulmonary spinal afferent TRPV1 denervation with RTX. These data describe the presence of a chemosensitive pulmonary sympatho‐excitatory reflex that can be stimulated by noxious stimuli in the lung. The present study showed that activation of this pulmonary spinal reflex can be evoked on either the dorsal or ventral lung surface, suggesting a wide pulmonary distribution of these chemosensitive afferents.

Activation of these spinal sensory nerve fibers in the lung increases efferent sympathetic nerve activity as evidenced by an increase in RSNA, which likely mediated the increase in HR and AP. Previous studies investigating the presence and physiological role of pulmonary spinal afferents have been inconclusive (Weaver 1977; Kostreva et al. 1981; Wang et al. 2003; Soukhova‐O'Hare et al. 2006; Hooper et al. 2016), and the distribution of spinal afferent neurons within the lung is unknown. Early studies indicated that tracheal afferent nerve fibers originated in the C1 and T1–T4 DRG (Dalsgaard and Lundberg 1984; Kummer et al. 1992) and that pulmonary spinal afferent activity can be recorded from T2 to T4 (Kostreva et al. 1981), the same upper thoracic segments in which cardiac sympathetic afferents pass (Wang et al. 2014). Consistent with the current study, a study by Qin et al. (2007) showed that noxious stimuli applied to both the lung and the heart altered activity within the same convergent T3 spinal neurons. In the present study, chemical stimulation of the ventral lung visceral pleura evoked similar pressor responses to that of cardiac afferent activation. This observation, considering the adjacent location between lung and heart, suggests that pulmonary chemosensitive spinal afferents and cardiac chemosensitive spinal afferents may be signaling through the same pathway. This concept is further supported by the fact that ablation of TRPV1 receptor‐expressing neurons/fibers in the upper thoracic DRG/spinal cord with RTX abolished both cardiac and pulmonary sympathetic afferent reflexes. Moreover, the different cardiovascular response seen with dorsal vs. ventral lung afferent activation suggests that nerves innervating the dorsal surface of the lung converge through a pathway separate to those innervating the ventral surface.

Previous studies have indicated a species‐ and fiber type‐dependent difference on the response to stimulation of upper thoracic spinal afferents. Electrical stimulation of the severed caudal postganglionic branch of the stellate ganglia in cats produced a depressor response, reducing AP and RSNA at low stimulation frequencies, and a pressor response when the same nerve was stimulated at high frequency (Weaver 1977). Furthermore, a later study by Kostreva et al. (1981) demonstrated that electrical stimulation of various afferent components in the stellate nerve branch, which receives innervation from both the heart and the lung, produced different cardiovascular effects in dogs and monkeys. These investigators observed that stimulation of cut stellate nerves with conduction velocities of 2.5–10 m/sec (in the range of A*δ* fibers) resulted in a depressor response, whereas stimulating afferent fibers with conduction velocities of 0.5–3.0 m/sec (C fibers) elicited a pressor response in the anaesthetized monkey and dog (Kostreva et al. 1981). These results were maintained after bilateral vagotomy. Taken together, these studies suggest that upper thoracic spinal afferents contain both sympatho‐excitatory (most likely metabolically sensitive C fibers) and sympatho‐inhibitory (most likely A fibers) components in many mammalian species. Therefore, it is reasonable to assume that spinal afferents in lungs contain sympatho‐excitatory fibers as well. Unfortunately, these studies did not verify the specific origin of these upper thoracic spinal afferents (i.e., heart vs. lung), so it is not possible to firmly conclude from these data that specific pulmonary sympatho‐excitatory afferents exist in the species used in these studies.

With regard to chemosensitive pulmonary spinal afferents, Soukhova‐O'Hare and colleagues had reported that the lung contained spinal chemically sensitive sympatho‐inhibitory afferent fibers in the rabbit (Wang et al. 2003; Soukhova‐O'Hare et al. 2006). In these studies, the authors found that direct injection of BK into lung parenchymal resulted in decreased AP, HR, and RSNA. No pressor or sympatho‐excitatory response was reported during lung parenchymal BK injection. In contrast to the findings by Soukhova‐O'Hare and colleagues, our study describes a potent renal sympatho‐excitatory and cardio accelerator responses to topical application of either BK or Cap onto the lung visceral pleura in anesthetized, vagotomized rats. On the other hand, we observed a predominantly depressor response to topical application of Cap onto the dorsal surface of the lung while HR and RSNA increased. These discrepancies of the present study from previously reported data are unclear. First, the studies of Soukhova‐O'Hare et al. described a depressor/sympatho‐inhibitory response to BK in anesthetized rabbits, where we report a pressor/sympatho‐excitatory response to BK in anesthetized rats. The differential response in magnitude to dorsal or ventral application of agonist for the data presented here indicates that dorsal and ventral surfaces of the lung may either contain different subpopulations of spinal afferents, a different density of afferent innervation or project to different central cardiovascular control regions. As BK primarily activates somatic/visceral C‐fiber afferents, it is likely that lungs in rabbits contain predominantly sympatho‐inhibitory C‐fiber afferents whereas lungs in rats contain sympatho‐excitatory C‐fiber afferents, although this cannot be confirmed. These discrepancies may could also be related to the different modes of application of BK to the lung parenchyma affecting access to different populations of chemosensitive spinal afferents. Our data are consistent with earlier findings in the dog, cat, and monkey, which suggest that upper thoracic spinal C‐fiber afferents are predominantly sympatho‐excitatory (Weaver 1977). We chose to topically apply BK onto the surface of lungs visceral pleura, whereas Soukhova‐O'Hare et al. injected BK into the lung parenchyma. Importantly, they did not clarify in detail which part of the lung BK was injected. As we documented that dorsal or ventral lung application of BK and Cap can cause differential cardiovascular responses, it is possible that injection of agonists into different areas of the lungs may contribute to the discrepancies between our results. Nevertheless, we observed a similar pressor/sympatho‐excitatory response with intrabronchial BK application, compared to topical application. Although the effects of intrabronchial application of BK may be similar to inhaled BK, they are not equivalent. The position of the intrabronchial cannula at the level of the bronchi may have prevented BK activation of upper airway receptors. Intrabronchial BK will likely stimulate receptors in the airways and lungs, as well as entering the circulation. Therefore, we conclude that rat lungs contain spinal sympatho‐excitatory afferents, similar to many other organs (i.e., heart, muscle, and gallbladder). To the best of our knowledge, this is the first study to identify that chemosensitive spinal sympatho‐excitatory afferents exist on or near the surfaces of the rat lung.

Capsaicin is a potent agonist of the TRPV1 receptor. Capsaicin‐sensitive neurons are predominantly thought to be unmyelinated C fibers; however, a subset of capsaicin‐sensitive A*δ* fibers has also been identified (Szallasi et al. 2007). The biphasic effect observed following capsaicin application in some of our experiments may be due to its activation of both A*δ* and C fibers on the lung surface. The thinly myelinated A*δ* fibers could be contributing to the observed depressor response, masking any initial pressor response seen by C‐fiber activation.

Conversely to the Cap binding to TRPV1, a nonselective cation channel, BK is thought to predominantly signal through the B_2_ receptor, a G_q_‐coupled GPCR, on sympathetic sensory neurons (Jones et al. 1995; Szallasi et al. 2007). However, there may also be low levels of expression of the B_1_ receptor (Vellani et al. 2004) which can modulate TRPV1 receptor neuronal activity (Szallasi et al. 2007; Mathivanan et al. 2016). The differential responses seen with BK and Cap application may be due to their mode of activation and the fiber type they are stimulating, although we cannot confirm the distribution and density of nerve fibers on the dorsal to ventral surface of the lung. Recent studies by the Liberles laboratory have used reporter viruses to map the vagal innervation of the lung (Chang et al. 2015). A technique similar to this would be useful to specifically map the sensory nerve fiber distribution and their fiber type within the lung. Another explanation for the differing responses of BK and Cap is that Cap may be more effective than BK at locally evoking release of tachykinins, neuropeptides, or additional neuromodulators or that Cap may directly modulate smooth muscle relaxation through K^+^ channels. This could be tested with direct inhibition of TRPV1 and using a variety of pharmacological techniques that were beyond the scope of this study.

In our preparation, the chest was opened in such a way that the ventral surface of the lung could be accessed without touching the heart, so as to reduce any confounding results from accidental activation of cardiac sympathetic afferents (Wang et al. 2017), as previously described. Although there is a very small possibility that some of the hemodynamic changes observed could have been due to absorption of BK or Cap through the lung into the circulation, the rapid onset of the response, the immediate increase in sympathetic nerve activity, and the immediate termination of the response upon removal of the agonists lead us to believe that the major component of HR and AP changes seen in this study was due to activation of an pulmonary autonomic reflex pathway. There is also a possibility that by activating afferent fibers with topical application of BK and Cap, that afferent nerves from pulmonary arteries or veins near the hilum may be activated and that these fibers would also directly innervate the heart. By applying the agonists to the midsection of the left lung, far from the hilum, we aim to avoid this. While Cap is a selective agonist for the TRPV1 receptor, BK activates sympathetic afferents by specific subtypes of the BK receptor (Soukhova‐O'Hare et al. 2006). Therefore, BK and Cap may be stimulating different subtypes of pulmonary spinal sensory fibers. However, the abolishment of the BK response post‐RTX treatment of the T1–T4 DRG indicates that the BK response was mediated through TRPV1‐positive nerve fibers.

Importantly, in this study, we confirmed the spinal origin of this pulmonary reflex by RTX‐induced ablation of spinal afferent ganglia. RTX, an ultrapotent analogue of Cap, is capable of inducing rapid degeneration of TRPV1‐expressing afferent neurons and fibers (Wang et al. 2014). Our recent study (Wang et al. 2014) demonstrated that epicardial application of RTX ablated TRPV1‐positive nerve fibers from the surface of the heart and blocked the cardiac sympathetic afferent reflex evoked by epicardial application of BK or Cap. In the current study, we performed epidural delivery of RTX to ablate the TRPV1‐positive DRG soma. This procedure markedly reduced the number of TRPV1‐positive sensory neurons in the thoracic DRG and prevented the pressor/sympatho‐excitatory reflex observed when BK or Cap was applied to the lung or the heart. These data strongly suggest that the pulmonary reflex observed in vagotomized animals is due to signaling through a spinal sympathetic afferent pathway rather than either vagal afferents or through a systemic circulatory effect. These data also confirm that the reflex is mediated by TRPV1 sensory afferents. The lack of any bradycardiac response observed in animals with intact vagi, as may be expected if BK or Cap were also activating vagal afferent C fibers, may be due to differential distribution of vagal and spinal afferents within the lung tissue and airway. Reflexes were only tested in animals with intact vagi with topical application of agonists to the visceral pleura. Specific vagal and spinal afferent labeling could establish the distribution and colocalisation of afferent nerve fibers within the airways (Chang et al. 2015) or vagal reflexes may be present but bradycardiac responses could have been masked by the potent tachycardia (Oh et al. 2006). Systemic administration of propranolol in vagal intact animals, prior to the application of BK or Cap, may unmask this.

The identification of a pulmonary sympatho‐excitatory reflex that responds to noxious stimuli may have important clinical implications. It is thought that most spinal sympathetic fibers are small, slow conducting C fibers, and that this fiber type is dormant in most physiological conditions. However, these fibers are often activated in conditions of inflammation and stress. A link between inhaled activators of pulmonary sensory neurons and altered cardiac function has previously been described; however, its vagal or spinal reflex origin is inconclusive (Hooper et al. 2016). Clinically, an increased prevalence of cardiovascular events in areas of high pollution is well documented (Pope et al. 2004; Mills et al. 2009; Brook et al. 2010). These risk factors are further increased if the patients have a history of cardiovascular disease (Peters et al. 2001; Mann et al. 2002). A potential influence of activation of pulmonary spinal afferents is their contribution to the increased risk of cardiovascular events and reduced prognosis in patients with existing disease. For instance, those suffering from chronic obstructive pulmonary disease (COPD) are more likely to have major adverse cardiovascular events from cardiovascular comorbidities than those without COPD (Calverley and Scott 2006; Álvarez et al. 2008; Zoccali et al. 2013; Ooi et al. 2018). A greater understanding of this pathway may contribute to the development of new therapeutic strategies. We believe that this pulmonary sympatho‐excitatory response will be helpful to further understand the role of pulmonary spinal sensory nerves in modulating cardiovascular function and autonomic activity in healthy and disease states.

## Conflict of Interest

None.
